# Attitudes towards body and perception of parental care and close relationships in anorexia nervosa (AN)

**DOI:** 10.1192/j.eurpsy.2022.1484

**Published:** 2022-09-01

**Authors:** P. Karpenko, L. Pechnikova, E. Zhuykova, E. Sokolova, A. Ryzhov, O. Ioannisyants

**Affiliations:** 1 Lomonosov MSU, Faculty Of Psychology, Moscow, Russian Federation; 2 Russian State University for the Humanities, L.s. Vygotsky Institute Of Psychology, Moscow, Russian Federation; 3 G. E. Sukharevascientific and practical center for mental health of children and adolescentsof the Moscow departament of Public Health, Division 5, Moscow, Russian Federation

**Keywords:** perceived parental care, Anorexia nervosa, attachment in close relationships, body image

## Abstract

**Introduction:**

The links between body image disturbances and distorted relationships with parents were supposed since the early conceptualizations of AN by Hilde Bruch. The empirical studies however were concerned with perceptual aspects of body image and much less is known about how the attitudinal aspects and the body-related behaviors are affected.

**Objectives:**

To study the attitudinal and behavioral aspects of body image in adolescents with AN in relation to perceived parental care and the attachments to close people.

**Methods:**

The Body Investment Scale (BSI), Parental Bonding Instrument (PBI) and Attachment Style Questionnaire (ASQ) were used. 53 girls with Anorexia Nervosa were compared to 63 controls (adjusted by age).

**Results:**

Girls with AN scored significantly higher on BIS Body attitude (p<.001) and Protection scales (p<.01), while displayed equal results on Body Care scale. They displayed lower Confidence in relationships (p<.01), higher Need for approval, Discomfort and preoccupation in close relationships (p<.05). No differences were found on PBI, excerpt for AN group perceiving less paternal control (p<.05). The correlation analysis, while showing a number of similar correlations within groups, suggests that in AN group positive Body image was more closely linked to perceived early care, especially from father (r=.6), in contrast with controls. In current relationships negative Body image for AN is stronger related to Discomfort and Need for approval (r>0.6), while Discomfort with Touch is less linked to problems in relationships than in controls.

**Conclusions:**

Results suggest the importance of studying the father’s mediating role in the formation of body attitudes in AN.

**Disclosure:**

No significant relationships.

